# First in man study of intravitreal tripeptidyl peptidase 1 for *CLN2* retinopathy

**DOI:** 10.1038/s41433-023-02859-4

**Published:** 2023-12-04

**Authors:** James Wawrzynski, Ana Rodriguez Martinez, Dorothy Ann Thompson, Dipak Ram, Richard Bowman, Rebecca Whiteley, Chin Gan, Louise Harding, Amanda Mortensen, Philippa Mills, Paul Gissen, Robert H. Henderson

**Affiliations:** 1 UCL Great Ormond Street Institute of Child Health, London, UK; 2 NIHR Biomedical Research Centre, Great Ormond Street Hospital, London, UK; 3 Manchester University NHS Foundation Trust, Manchester, UK; 4 Batten Disease Family Association, Shipley, UK

**Keywords:** Drug therapy, Retinal diseases

## Abstract

**Background/Objectives:**

CLN2 Batten Disease is a fatal neurodegenerative condition of childhood associated with retinal dystrophy and blindness. Intracerebroventricular infusion of rhTPP1 greatly slows the rate of neurodegenerative decline but not retinopathy. Intravitreal rhTPP1 is known to slow retinal degeneration in a canine model of CLN2. We report a first-in-man controlled clinical trial of intravitreal rhTPP1 for CLN2 associated retinal dystrophy.

**Subjects/methods:**

8 children aged 5–9 with CLN2 Batten Disease were prospectively enroled. Severely affected patients were preferentially selected, provided that vision was better than no perception of light. Children underwent 8 weekly intravitreal injections of rhTPP1 (0.2 mg in 0.05 ml) into the right eye for 12–18 months. The left eye was untreated and acts as a paired control. The primary outcome was safety based on the clinical detection of complications. A secondary outcome was paracentral macular volume (PMV) measured by spectral domain OCT. Linear regression/paired t tests were used to compare rates of decline.

**Results:**

No severe adverse reactions (uveitis, raised IOP, media opacity) occurred. The mean baseline PMV was 1.28 mm^3^(right), 1.27 mm^3^(left). 3 of the youngest patients exhibited bilateral progressive retinal thinning (*p* < 0.05), whereas retinal volume was stable in the remaining 5 patients. In the 3 patients undergoing retinal degeneration, the rate of PMV loss was slower in the treated vs. untreated eye (*p* = 0.000042, *p* = 0.0011, *p* = 0.00022).

**Conclusions:**

Intravitreal rhTPP1 appears to be a safe and effective treatment for CLN2 related retinopathy however commencement of treatment early in the course of disease is more likely to be efficacious.

## Introduction

Batten disease (neuronal ceroid lipofuscinosis) is a collective term for a genetically heterogeneous group of neurodegenerative conditions. They are characterised by a period of normal neurological development after birth followed by rapid regression, with the timing of onset dependent on the subtype of disease. Infantile, late infantile, juvenile and adult forms are recognised [1]. Disease occurs due to dysregulation of lysosomal protein catabolism, causing the accumulation of auto-fluorescent lipofuscin-like material within the lysosomes of neuronal cells and ultimately resulting in atrophy within the central nervous system and neural retina.

There are 13 known disease-causing genes for Batten disease and inheritance is predominantly autosomal recessive [1]. Pathogenic variants within each gene tend to cause disease in a highly stereotyped manner. There is no FDA/EMA approved disease modifying treatment for any form of Batten disease, with the exception of Neuronal Ceroid Lipofuscinosis type 2 (CLN2), for which the intracerebroventricular drug cerliponase alfa was approved in 2017 [2].

CLN2 is a form of late infantile Batten disease. The age of onset is typically between the ages of 2–4. Patients initially develop seizures followed by progressive motor and speech decline, worsening vision and premature death by the middle of the second decade [3]. Vision loss occurs secondary to retinal dystrophy, which is characterised by progressive outer retinal degeneration beginning in the foveal ellipsoid zone, bulls eye maculopathy, vessel attenuation, optic disc pallor and optic nerve thinning [4]. CLN2 is caused by bi-allelic loss of function mutations in *CLN2* (11p15.4), encoding lysosomal tripeptidyl peptidase 1 (TPP-1). Although 140 pathogenic variants have been reported, just two account for the majority of cases (c.622 C > T (Arg208*) and c.509–1 G > C (splice site mutation)) [1]. TPP-1 is responsible for cleaving tripeptides from the N-termini of proteins within the lysosomes of neuronal cells. Reduced TPP-1 activity results in defective protein degradation causing an accumulation of ceroid lipofuscin, resulting in widespread loss of neural tissue.

Cerliponase alfa (recombinant human TPP-1 enzyme replacement therapy – ERT) was licenced for the treatment of neurological regression associated with CLN2 in 2017. It is administered by 2-weekly intracerebroventricular (ICV) infusion and has been shown to slow down progression of neurological disease and cerebral volume loss [2]. ICV ERT does not, however, prevent CLN2 retinal dystrophy.

We hypothesise that the reason ICV ERT is not an effective treatment for CLN2 retinal dystrophy is because it does not cross the blood brain barrier and therefore does not have access to the vitreous or retina. Consistent with this hypothesis is recent evidence that regular intravitreal injections of ERT (IVT ERT) in combination with ICV ERT, but not ICV ERT alone, can rescue retinal dystrophy in a canine model of the disease [5]. We therefore designed a first-in-man study of the feasibility and safety of IVT ERT in human CLN2 patients to test the hypothesis that IVT ERT could slow down the progression of retinal dystrophy in CLN2 patients also receiving ICV ERT.

## Methods

### Study design/participants

A prospective interventional controlled compassionate use study was designed at a single centre to investigate the safety and efficacy of IVT ERT in CLN2 retinopathy. The study was open-label and non-randomised. Inclusion criteria were: patients had a diagnosis of CLN2 confirmed by mutation analysis and TPP1 enzyme assay; presence of advanced CLN2 retinopathy but vision better than no perception of light; current treatment with ICV ERT. The study was funded by the Batten Disease Family Association and had a capacity to enrol a maximum of 8 patients. Eight severely affected patients [4] who best satisfied the inclusion criteria were therefore selected. Written informed consent was received from parents/guardians prior to participation. Data collection was approved by the national research ethics service in the UK (national research ethics service committee: London – Bloomsbury, REC reference 13/LO/0168) and the study was performed in compliance with the tenets of the declaration of Helsinki.

### Protocol

Patients underwent a baseline ERG/VEP, MRI head, supine macular OCT (Heidelberg Spectralis FLEX, Heidelberg Engineering Ltd), RetCam imaging of the fundus in 9 positions, IOP check and examination under anaesthesia. ERG results were converted into an ERG score as described previously [6]. OCT scans were manually segmented at the RPE and ILM to measure retinal thickness. Segmented images were used to calculate the paracentral macular volume (PMV), defined as the macular volume within a fovea-centred circle of 3 mm diameter, excluding the central 1 mm. RetCam images and OCT scans were used to calculate the severity of retinopathy at enrolment based on the neuronal ceroid lipofuscinoses (NCL) ophthalmic severity score [4].

Patients received 12–18 months of 8-weekly IVT ERT (0.2 mg rhTPP-1 in 0.05 ml) into the right eye under general anaesthesia (GA). IVT ERT was prepared using overage from brineura sourced for ICV ERT in the same patients (method described in supplementary material). This dose was chosen by proportionally scaling up from the 0.1 mg rhTPP-1 used previously in a canine study [5], assuming the human vitreous volume to be approximately double that of the dog. Following each injection, the patency of the central retinal artery was checked and an anterior chamber paracentesis was performed if occluded. Post procedural topical dexamethasone was prescribed QDS for 4 weeks since uveitis is a known risk from the animal study. Topical chloramphenicol was also prescribed QDS for 1 week after each injection.

The left eye acted as an untreated paired control in all cases. The EUA, IOP and supine OCT’s were repeated under GA prior to each injection.

### Outcome measures

1. The primary outcome was the safety of IVT ERT, based on the clinical detection of ocular complications. Patients were monitored for cataract, uveitis and elevated intraocular pressure (IOP) - defined as IOP > 21 mmHg or an IOP > 5 mmHg higher in the treated vs. untreated eye.
2. The secondary outcome was efficacy of IVT ERT based on the PMV. PMV was chosen because retinal degeneration in CLN2 Batten disease is known to affect the central macula first. We excluded the subfoveal retinal thickness from measurements because we enroled severely affected patients with significant/end stage subfoveal degeneration.

### Statistics

R was used to perform linear regression in order to estimate rates of decline in treated versus untreated eyes. Data were tested for consistency with a normal distribution (Shapiro-Wilk test). Data relating to PMV and rates of change in PMV were found to be normally distributed (*p* > 0.05) and therefore paired two-sided t tests were used to compare these rates of decline between the left and right eyes of each patient over the course of the study. *p* < 0.05 was considered statistically significant. Data relating to ERG scores were analysed with the Wilcoxon Signed Rank Test.

## Results

Eight patients (4 M and 4 F) with a median age of 7.5 years (range 5–10) were enroled. All patients completed between 12 to 18 months of follow-up with IVT ERT at approximate 2 monthly intervals. Two patients required their injections to be postponed by three weeks on one occasion each as they were required to isolate due to COVID-19 infection.

Individual patient characteristics, together with patients’ ages and CLN2 scores at the time of ICV ERT commencement and at the time of IVT ERT commencement are detailed in Table 1. The most common pathogenic variant in *CLN2* in our cohort was c.509–1 G > C. At the start of IVT infusions the mean NCL ophthalmic score was 4.38, the mean PMV was 1.28mm^3^ (right), 1.27mm^3^ (left) and the mean ERG score was 0.37 (right) and 0.40 (left). There was no statistically significant difference between the baseline PMV or ERG score of left versus right eyes (*p* > 0.05). MRI brain scans showed predominantly subcortical degeneration with associated symmetrical degeneration of the optic nerves.

**Table 1:** Baseline characteristics of enroled patients.

Patient number	CLN2 variant(s)	Age at first ICV infusion	CLN2 score at first ICV infusion	Age at first IVT	CLN2 score at first IVT	Mean ERG score before enrolment	NCL ophthalmic score at first IVT
1	c.509–1 G > C c.509–1 G > C	4 y, 7 m		5 y, 4 m		0	4
2	c.509–1 G > C c.509–1 G > C	4 y, 7 m	2 + 2	6 y, 2 m	0 + 1	0.6	5
3	c.622 C > T c.1094 G > A	4 y, 5 m	2 + 2	6 y, 4 m	2 + 2	1	3
4	c.622 C > T c.1678–1679 del	4 y, 10 m	1 + 1	6 y, 8 m	1 + 1	0.8	3
5	c.509–1 G > C c.509–1 G > C	3 y 9 m	3 + 3	8 y, 4 m	2 + 2	0.2	5
6	c.1052 C > T c.1052 C > T	4 y, 5 m	2 + 0	8 y, 6 m	1 + 0	0	5
7	c.509–1 G > C c.509–1 G > C	4 y, 8 m	2 + 1	9 y, 4 m	0 + 0	0.2	5
8	c.754–757 del c.1094 G > A	3 y, 7 m	3 + 2	10 y, 4 m	2 + 2	non-compliant - no data	5

At 2-monthly examinations under anaesthesia, there were no instances of uveitis, media opacity, raised IOP or other iatrogenic ocular pathology. Central retinal artery occlusion occurred momentarily after IVT in two patients who had pre-existing severe vessel attenuation and was alleviated by anterior chamber paracentesis. One patient experienced laryngospasm related to general anaesthesia and missed one fortnightly ICV ERT infusion. A large subconjunctival haemorrhage occurred in one patient after his first intravitreal injection. Further investigation revealed idiopathic thrombocytopenia and he was subsequently treated with peri-procedural tranexamic acid, which prevented further occurrences of large subconjunctival haemorrhage.

**Fig. 1:**
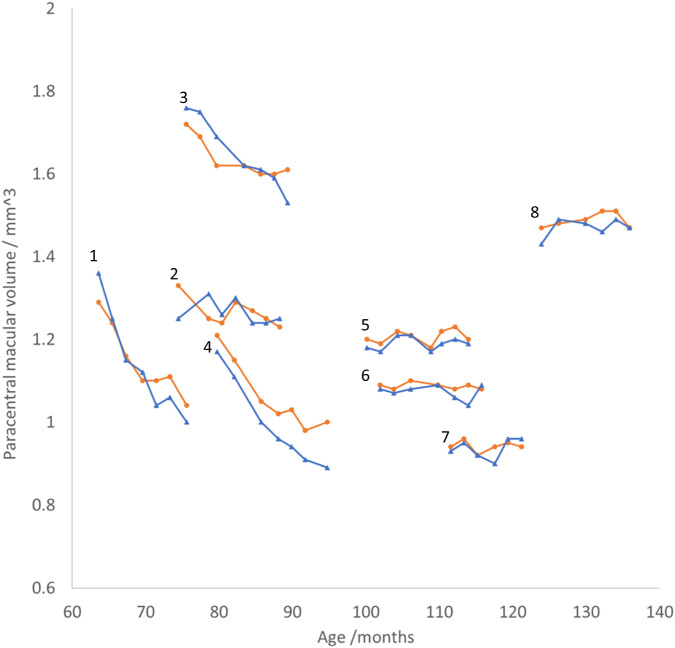
PMV for each eye of every patient is plotted against age. Lines connect points corresponding to the same eye. Right eyes -orange dots. Left eyes - blue triangles. Each patent is numbered in accordance with their designated ID in Fig. 2.

**Fig. 2:**
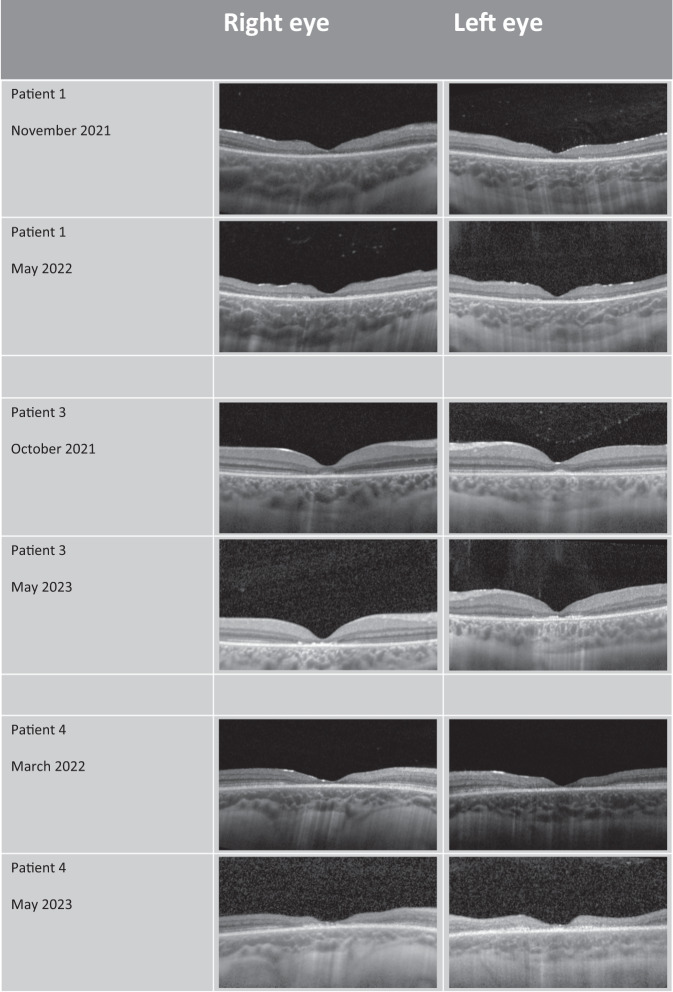
OCT images showing the progression of CLN2 related retinal thinning in the three patients who were in the active/progressive phase of CLN2 retinopathy over the course of the study. In each case, patients underwent 2-monthly intravitreal injections of TPP-1 in the right eye. In each case, the outer retina in the left eye has thinned more than in the right eye.

Over the study period, a statistically significant rate of PMV reduction was found in both eyes of three patients (right eyes; *p* = 0.012, *p* = 0.0017, *p* = 0.0028, left eyes; *p* = 0.000042, *p* = 0.0011, *p* = 0.00022) but in neither eye of the remaining 5 patients (*p* > 0.05). Standard deviations were similar between right and left eyes (right: 0.00761mm^3^/month, left: 0.0111 mm^3^/month) and in each case data were normally distributed. The mean age at enrolment of patients in whom a PMV reduction occurred was 6 yrs, 1mo whereas the mean age at enrolment in the stable PMV group was higher at 8 yrs, 6mo (*p* = 0.021). The mean PMV at baseline in patients who experienced retinal thinning was 1.42mm^3^, whereas the mean PMV at baseline in patients who did not was lower at 1.19mm^3^ (*p* = 0.029). In patients who experienced bilateral PMV decline over the study period, the mean rate of decline was 0.168mm^3^/year in treated (right) eyes and 0.254mm^3^/year in untreated (left) eyes. In all 3 cases, the rate of decline was lower in right eyes than left eyes and this difference was a statistically significant; *p* = 0.015, *p* = 0.022, *p* = 0.0050. Complete PMV data are shown in Fig. 1 and values at treatment initiation and at the end of the study are shown in Table 2. An example from one patient, who showed less thinning in the right eye compared to the left eye is shown in Fig. 2.

**Table 2:** OCT and ERG measurements before and after treatment.

Patient number	Right eye PMV (pre-treatment)/mm^3^	Right eye PMV (final value post-treatment)/mm^3^	Left eye (pre-treatment)/mm^3^	Left eye (final value post-treatment)/mm^3^	Right eye ERG score (pre-treatment)	Right eye ERG score (after treatment initiation)	Left eye ERG score (pre-treatment)	Left eye ERG score (after treatment initiation)
1	1.29	1.04	1.36	1.00	0	*not recorded*	0	*not recorded*
2	1.33	1.23	1.25	1.25	0.5	0	0.5	0
3	1.72	1.61	1.76	1.53	1.0	*not recorded*	1.0	*not recorded*
4	1.21	1.00	1.17	0.89	0.6	0.4	0.9	0.4
5	1.20	1.20	1.18	1.19	0.3	*not recorded*	0.1	*not recorded*
6	1.09	1.08	1.08	1.09	0	*not recorded*	0	*not recorded*
7	0.94	0.94	0.93	0.96	0.2	*not recorded*	0.2	*not recorded*
8	1.47	1.47	1.43	1.47	*not recorded*	*not recorded*	*not recorded*	*not recorded*

**Fig. 3:**
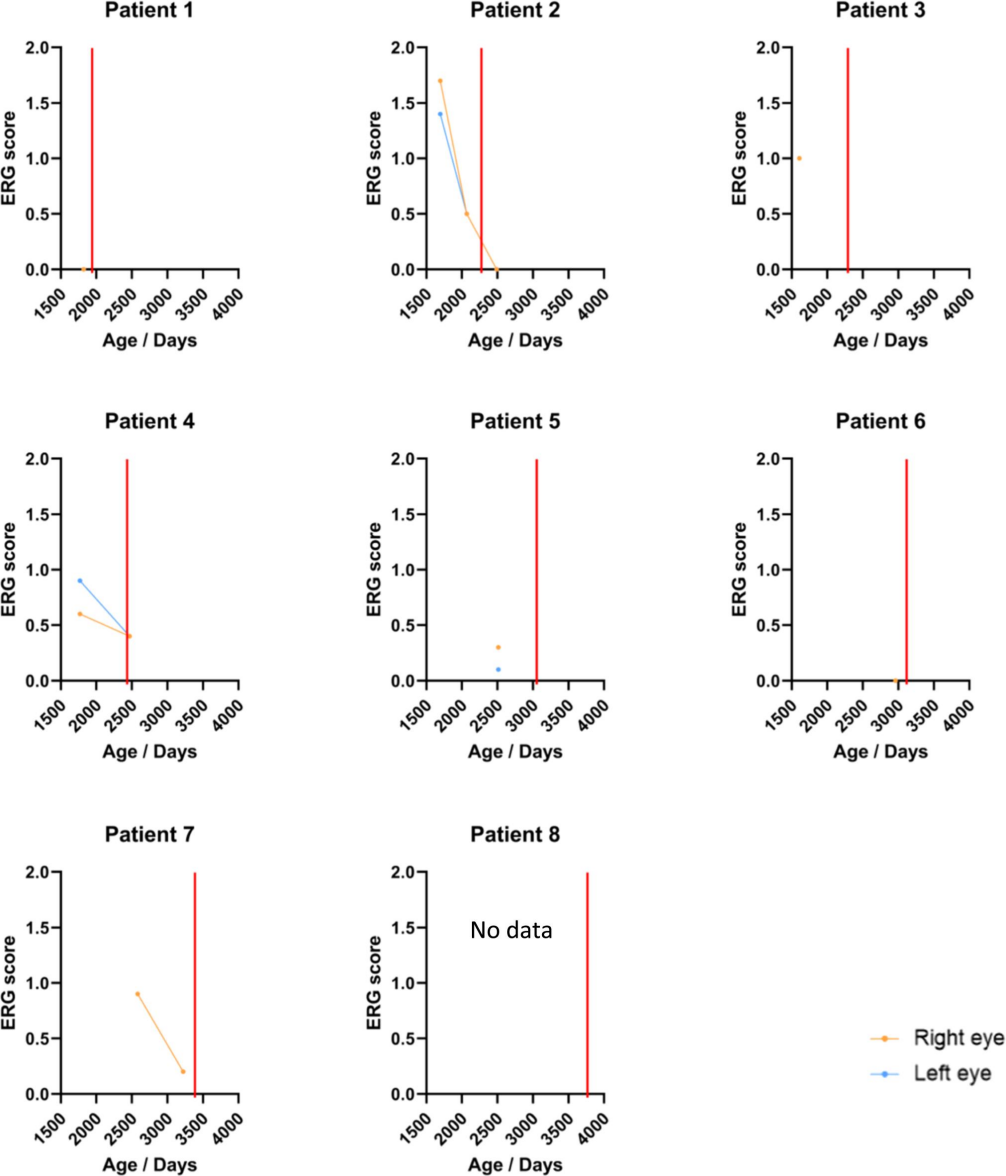
ERG scores by patient over time. The age at first intravitreal injection of rhTPP-1 is indicated in each case by a vertical red line.

Seven patients underwent electroretinography at baseline, before initiation of treatment. However, only two patients successfully completed ERG testing at any time after initiation of treatment. No benefit of treatment has thus far been found with respect to preservation of ERG scores (Fig. 3, Table 2). All patients underwent baseline MRI head before initiation of treatment however the protocol used did not offer sufficient resolution of the optic nerves to quantify optic nerve degeneration. One patient who had the largest treatment effect with respect to the difference between left and right eye retinal degeneration underwent high resolution MRI orbits after one year of treatment. In this patient, both optic nerves had undergone significant degeneration at baseline and no difference was found between the calibre of the left and right optic nerve after 1 year of treatment.

## Discussion

In our study, we demonstrated the feasibility of the use of overage of ICV ERT infusions for IVT ERT injections.

IVT ERT was a well-tolerated treatment for CLN2 related retinal dystrophy, which showed a statistically significant treatment effect even in patients approaching end stage CLN2 retinopathy.

### Safety

During the study period, no serious ocular complications related to the treatment were detected. No previous literature describing IVT ERT in human patients exists, however in a previous study investigating the use of IVT ERT in dogs, severe anterior and intermediate uveitis developed. This raised a concern about possible pro-inflammatory properties of the drug. In our study, a careful slit lamp examination at each examination under anaesthesia did not detect any evidence of uveitis in human patients, suggesting that the inflammation seen in the canine model may be related to the use of xenogeneic (human) TPP-1 in the canine study.

Two patients with the most severe vascular attenuation required the creation of an anterior chamber paracentesis following intravitreal injections in order to reduce the intraocular pressure since CRA occlusion was noted on indirect ophthalmoscopy immediately following intravitreal injections. Further work is required to understand whether immediate paracentesis is mandatory or not in such patients.

One systemic adverse event occurred in a patient who experienced laryngospasm following general anaesthesia. Anaesthetic complications highlight the importance of balancing any benefits of ocular treatment with IVT ERT against the risks of regular general anaesthesia. The possibility of performing intravitreal injections in this group of patients under sedation rather than general anaesthesia should also be considered in order to reduce anaesthetic risks.

### Efficacy

This is the first study to report a statistically significant treatment effect of IVT ERT on the rate of macular volume loss in patients with CLN2 retinal dystrophy. Macular volume loss in CLN2 retinopathy is known to follow a reverse s-shaped curve, being stable in early life, followed by a rapid decrease between the ages of 4 and 7 and finally reaching a plateau once end stage disease is reached [7]. Since the study was designed to focus on safety, patients with very advanced CLN2 retinopathy were selected for treatment. Accordingly, 5 of 8 patients enroled had reached end stage degeneration prior to enrolment and serial OCT scans did not reveal any further volume change in either eye during the study period. 3 of 8 patients were in the actively degenerating phase of disease. IVT ERT was effective at reducing the rate of macular volume loss in all 3 patients who were in the progressive/actively degenerative phase of retinal thinning, although the treatment effect was modest. However, for patients in end stage disease, IVT ERT neither positively nor negatively influenced the macular volume.

Whilst the presence of a statistically significant treatment effect is encouraging, further work will be required to determine whether modification of the treatment protocol or the timing of intervention with respect to patients’ age can improve the efficacy.

#### Age at treatment initiation

The mechanism of action of TPP-1 is by restoring lysosomal protein catabolism in the neural cells of the retina, which in turn prevents cell death. It is, however, not a reparative treatment. Although three of eight children in our study had not yet reached end stage disease at enrolment, they did all have advanced disease. Previous work investigating the effects of ICV ERT on cerebral volume loss shows that rapid volume loss may persist for one year after initiation of treatment [2]. It is likely that treatment earlier in the course of disease will deliver higher efficacy. CLN2 is usually diagnosed before retinal degeneration begins and therefore there is a potential therapeutic window between diagnosis and the onset of retinal degeneration during which IVT ERT may prove more efficacious.

#### Dose and frequency

The dose of rhTPP-1 used in this study was chosen to replicate the dose that was found to be effective in the canine study. This was done by increasing the dose in proportion to the increased vitreous volume in humans as compared to dogs. However, the dogs were treated before the onset of degeneration and therefore the degree of lipofuscin deposition in the dog retinas as compared to our patients was likely to have been much lower. The retention of TPP-1 within neuronal lysosomes is related to the concentration of lipofuscin, therefore in patients with advanced disease, a loading dose at treatment initiation may be beneficial. Since no adverse effects of TPP-1 were found in this study, future study design should consider the use of a loading dose.

The frequency of IVT ERT was every two months in this study, compared to 6 weekly in the canine study. The frequency was reduced to 2 monthly in order to prioritise safety and the treatment burden of regular general anaesthesia however we did not increase the treatment dose to compensate for this. Since loss of rhTPP1 from lysosomes occurs at an approximately constant rate that is related to lipofuscin concentration and independent of rhTPP1 concentration [8] future studies could maintain an 8-weekly interval whilst ensuring the presence of a therapeutic concentration of TPP-1 by proportionately increasing the treatment dose per intravitreal injection.

#### Route of administration

Although intravitreal ERT was highly efficacious in the dog study, canine and human CLN2 retinopathy have very different phenotypes. In the canine model, inner retinal degeneration prevails, causing an electronegative ERG [5], whereas in humans outer retinal degeneration and a reduced ‘a’ wave are seen [4, 6]. Therefore, the intravitreal route may be less suited to human disease. Changing the route of administration to subretinal is not feasible as this approach would require repeated pars plana vitrectomy. It may however be possible to increase the concentration of TPP-1 in photoreceptors by administration of a higher dose intravitreally. It is unlikely that the inner retinal layers would act as an absolute barrier that would prevent TPP-1 from accessing the outer retina, since transport through the retinal layers is known to take place by mannose 6-phosphate dependent uptake and transcytosis [9].

Further work should also consider optimising the study design and addressing which outcome measures best reflect any potential benefit of treatment. Previous literature has suggested that CLN2 retinal dystrophy is a highly symmetrical condition [7]; our results confirm this. Therefore, a paired comparison between right (treated) and left (untreated) eyes should continue to be used in order to reduce the sample size required when investigating this rare disease. Our results show that paracentral macular volumes are robust and repeatable measurements, which are likely to be able to detect small differences between treated and untreated eyes. On the contrary, the use of optic nerve MRI did not detect any treatment effect in this group of patients with advanced disease but would be likely to be an important anatomical outcome if treating patients before the onset of retinopathy/optic neuropathy. In addition to anatomical outcomes, functional outcomes will also be required. In the present study, visual acuities are not reported because the patients’ neurological regression precluded them from engaging reliably in testing and because their retinal disease was very advanced at baseline. Similarly, patients’ ERG traces were already highly attenuated at baseline and therefore ERG evidence of a functional treatment effect was limited. In a future study investigating the effect of early treatment with IVT ERT in children who had been on ICV ERT since diagnosis, both visual acuity and ERG scores are likely to be important functional outcomes.

In conclusion, this study is the first to demonstrate the feasibility and safety of IVT ERT in human patients with CLN2 retinopathy and to demonstrate a treatment effect. The data gathered pave the way for a larger trial earlier in the course of disease, before the development of retinal thinning. Since IVT ERT is well tolerated, treatment could be commenced with a high loading dose to ensure a therapeutic drug concentration from the moment of initiation and continued with a maintenance dose higher than that used in the present study in order to ensure a therapeutic concentration is achieved in the outer retina. Initiating treatment in patients at an early stage of disease would allow the collection of high quality functional as well as anatomical data.

Supplemental material is available at Eye’s website

## Summary

### What was known before

- Intravitreal rhTPP1 is known to slow retinal degeneration in a canine model of CLN2. We report a first-in-man controlled clinical trial of intravitreal rhTPP1 for CLN2 associated retinal dystrophy.

### What this study adds

- Intravitreal rhTPP1 is likely to be a safe and effective treatment for CLN2 related retinopathy however commencement of treatment early in the course of disease is important.

### Supplementary information

Standard operating procedure for the preparation of intravitreal rhTPP-1 from Brineura overage

## Data Availability

The datasets generated during and/or analysed during the current study are available from the corresponding author on reasonable request.

## References

[1] Mole SE, Cotman SL (2015). Genetics of the neuronal ceroid lipofuscinoses (Batten disease). Biochimica et Biophysica Acta (BBA)-Mol Basis Dis.

[2] Schulz A, Ajayi T, Specchio N, de Los Reyes E, Gissen P, Ballon D (2018). Study of intraventricular cerliponase alfa for CLN2 disease. N. Engl J Med.

[3] Nickel M, Simonati A, Jacoby D, Lezius S, Kilian D, Van de Graaf B (2018). Disease characteristics and progression in patients with late-infantile neuronal ceroid lipofuscinosis type 2 (CLN2) disease: an observational cohort study. Lancet Child Adolesc Health.

[4] Orlin A, Sondhi D, Witmer MT, Wessel MM, Mezey JG, Kaminsky SM (2013). Spectrum of ocular manifestations in CLN2-associated batten (Jansky-Bielschowsky) disease correlate with advancing age and deteriorating neurological function. PLoS One.

[5] Whiting RE, Pearce JW, Vansteenkiste DP, Bibi K, Lim S, Kick GR (2020). Intravitreal enzyme replacement preserves retinal structure and function in canine CLN2 neuronal ceroid lipofuscinosis. Exp Eye Res.

[6] Thompson DA, Handley SE, Henderson RH, Marmoy OR, Gissen P (2021). An ERG and OCT study of neuronal ceroid lipofuscinosis CLN2 Battens retinopathy. Eye.

[7] Kovacs KD, Patel S, Orlin A, Kim K, Van Everen S, Conner T (2020). Symmetric age association of retinal degeneration in patients with CLN2-associated batten disease. Ophthalmol Retin.

[8] Kim A, Grover A, Hammon K, de Hart G, Slasor P, Cherukuri A (2021). Clinical pharmacokinetics and pharmacodynamics of cerliponase alfa, enzyme replacement therapy for CLN2 disease by intracerebroventricular administration. Clin Transl Sci.

[9] Loh YP Mechanisms of intracellular trafficking and processing of proproteins. CRC Press; 1992.

