# Supplementary material for: First in man study of intravitreal tripeptidyl peptidase 1 for CLN2 retinopathy
Source: Eye (Lond). 2023 Dec 4;38(6):1176–82. doi: 10.1038/s41433-023-02859-4 (PMC11009280; doi:10.1038/s41433-023-02859-4)
Supplement: Supplementary file 1 — Standard operating procedure for the preparation of intravitreal rhTPP-1 from Brineura overage [file 41433_2023_2859_MOESM1_ESM.pptx]

## Slide 1
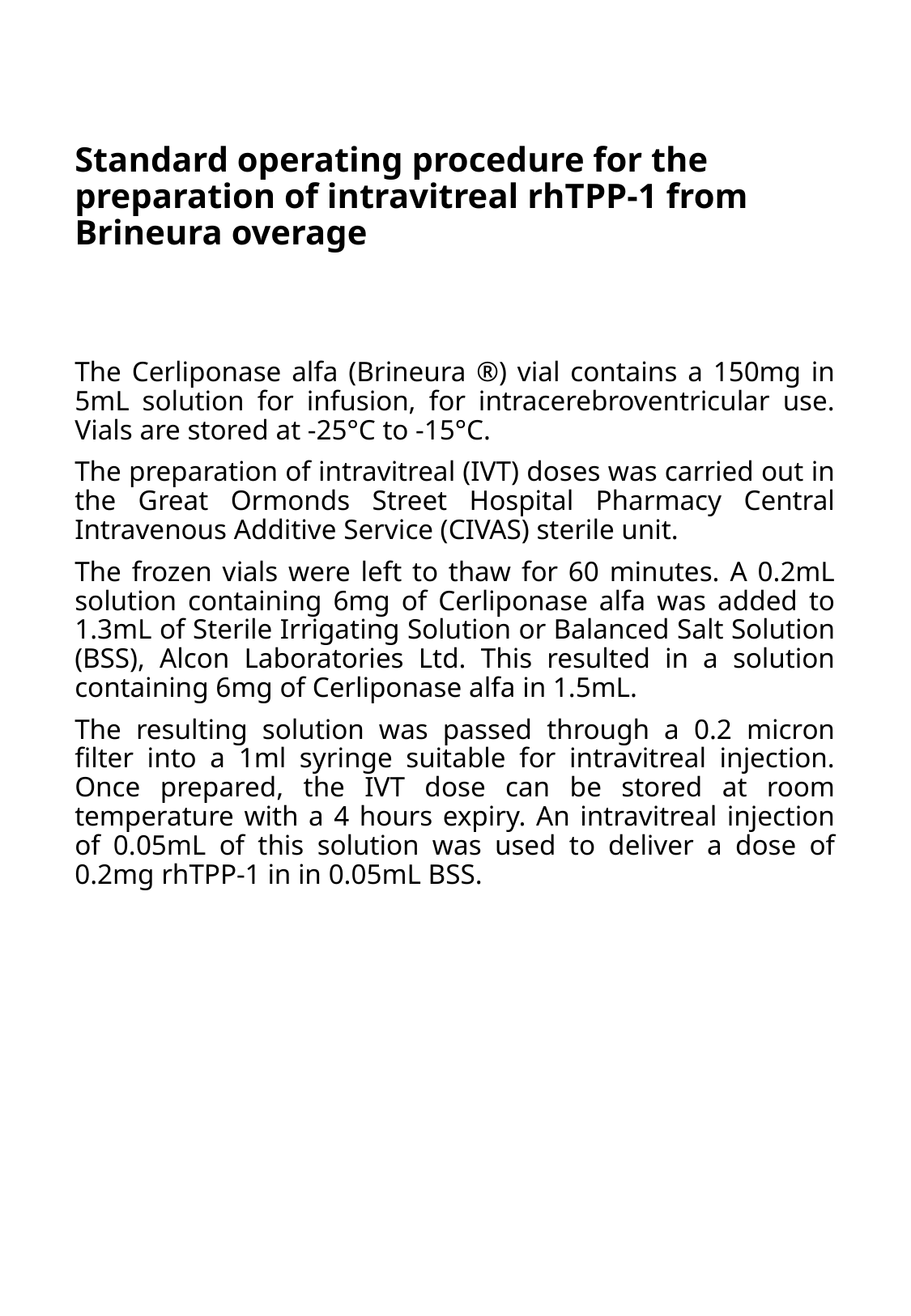

# Standard operating procedure for the preparation of intravitreal rhTPP-1 from Brineura overage
The Cerliponase alfa (Brineura ®) vial contains a 150mg in 5mL solution for infusion, for intracerebroventricular use. Vials are stored at -25°C to -15°C.
The preparation of intravitreal (IVT) doses was carried out in the Great Ormonds Street Hospital Pharmacy Central Intravenous Additive Service (CIVAS) sterile unit.
The frozen vials were left to thaw for 60 minutes. A 0.2mL solution containing 6mg of Cerliponase alfa was added to 1.3mL of Sterile Irrigating Solution or Balanced Salt Solution (BSS), Alcon Laboratories Ltd. This resulted in a solution containing 6mg of Cerliponase alfa in 1.5mL.
The resulting solution was passed through a 0.2 micron filter into a 1ml syringe suitable for intravitreal injection. Once prepared, the IVT dose can be stored at room temperature with a 4 hours expiry. An intravitreal injection of 0.05mL of this solution was used to deliver a dose of 0.2mg rhTPP-1 in in 0.05mL BSS.
